# Cell Structure Segmentation in TEM Images of Murine Skin Melanoma Cells by Deep Learning Model

**DOI:** 10.3390/jimaging12050215

**Published:** 2026-05-18

**Authors:** Mikhail A. Genaev, Izabella S. Gogaeva, Iuliia S. Taskaeva, Nataliya P. Bgatova, Mikhail V. Kozhekin, Evgeniy G. Komyshev, Dmitry A. Afonnikov

**Affiliations:** 1 Federal Research Center, Institute of Cytology and Genetics SB RAS, Novosibirsk 630090, Russia; 2 Kurchatov Center for Genome Research, Institute of Cytology and Genetics SB RAS, Novosibirsk 630090, Russia; 3 Research Institute of Clinical and Experimental Lymphology, Branch of the Institute of Cytology and Genetics, Siberian Branch of the Russian Academy of Sciences, Novosibirsk 630117, Russia; 4 Faculty of Natural Sciences, Novosibirsk State University, Novosibirsk 630090, Russia

**Keywords:** transmission electron microscopy, image segmentation, deep learning, neural network, mitochondria, endoplasmic reticulum, mitochondria–endoplasmic reticulum contact sites

## Abstract

Mitochondria–endoplasmic reticulum contact sites (MERCs) are known as the specialized areas that are involved in a large number of intracellular signaling pathways that regulate Ca^2+^ homeostasis, lipid transport, mitochondrial dynamics, cell death, and autophagy. Understanding MERC dynamics has important therapeutic implications in cancer, as these contacts regulate fundamental cellular processes and MERCs represent promising targets for therapeutic interventions aimed at improving cancer treatment outcomes. Despite the accumulated data, the role of MERCs in carcinogenesis still remains unknown; thus, it seems promising to search for new tools facilitating the study of MERCs in tumor cells. The structure of MERCs can be examined in great detail using transmission electron microscopy (TEM). Currently, several hundred TEM images are required to obtain reliable data on these contacts. The speed of data processing can be significantly improved by using fast and accurate image analysis techniques based on deep learning models. In this study, five U-Net models with a ResNet34 encoder network were evaluated, including the basic U-Net-Vanilla architecture as well as models incorporating various attention blocks and blocks capturing multilevel image structure, for the segmentation of mitochondria and the endoplasmic reticulum (ER). The best performance on the test dataset was demonstrated by the U-Net-scSE network, with *F*1 scores of 0.872 for mitochondria and 0.744 for the ER being achieved. Two models were tested for their ability to leverage pre-training on external datasets (Lucchi++, Kasthuri++, and DeepPi-EM). Additionally, models pre-trained on the CEM500K dataset were evaluated after the parameters had been tuned on the data. It was demonstrated by the results that pre-training or the use of pre-trained networks did not lead to an improvement in the *IoU* and *F*1 metrics on the test dataset. Subsequent image analysis was conducted to assess two types of MERCs in the segmented images. Finally, the free and user-friendly UltraNet web server was developed for automated analysis of mitochondria, ER, and MERCs using TEM images.

## 1. Introduction

The endoplasmic reticulum (ER) represents the cellular structure that is responsible for protein synthesis and folding, as well as for the biosynthesis of multiple lipids. The ER is composed of continuous membrane structures organized into distinct subdomains. Its architecture is dynamically remodeled in response to cellular needs through processes such as membrane fusion, fission, elongation, and degradation. The rough ER mainly consists of cisternae associated with ribosomes for protein synthesis, whereas the smooth ER predominantly comprises tubules involved in phospholipid biosynthesis.

Interactions between the ER and other organelles, particularly mitochondria, are essential for their function. The most extensively studied inter-organellar contact between the ER and mitochondria is termed the mitochondria–ER contact site (MERC), characterized by an intermembrane distance of 10 to 80 nm [1,2]. MERCs play critical roles in numerous intracellular signaling pathways regulating calcium (Ca^2+^) homeostasis, lipid transport, mitochondrial dynamics, cell death, and autophagy [1].

The functions of MERCs are also significant in various cancer cell types. Depending on the cancer type and stage, oncogenes and tumor suppressors localized at MERCs can exert diverse effects on ER–mitochondrial Ca^2+^ transport, thereby initiating either antitumor or pro-oncogenic outcomes [3]. MERCs serve as hubs for lipogenic and phospholipid synthesis pathways and are sites of reactive oxygen species (ROS) production in tumor cells, contributing to cancer cell death and fate determination [3,4,5]. Despite increasing evidence, the role of MERCs in carcinogenesis remains incompletely understood. Consequently, the development of novel tools to facilitate detailed studies of MERCs in tumor cells is a promising area of research.

Transmission electron microscopy (TEM) is one of the methods used to study cell ultrastructure, including MERCSs [6]. It allows for visual determination of the overall structures of cell organelles based on TEM images. Quantifying changes in organelle morphology requires additional software. There is a semi-automated approach for analyzing TEM images, based on the use of the *ImageJ* tool, also known as *Fiji* [7]. To obtain more reliable statistical estimates, the analysis of up to several hundred TEM images is required. In this case, even using a semi-automated approach is labor-intensive. It is possible to speed up data processing and increase its accuracy by using automatic image analysis systems.

The main task that has to be solved in the analysis of TEM images is semantic segmentation and morphometry of the identified objects [8,9,10,11,12]. To solve it, algorithms of image analysis [8,13,14,15], machine learning [9,16,17], and, more recently, deep learning are used [18,19,20]. For the analysis of biomedical images, among a variety of architectures, the U-Net architecture [21] is the most widely used. Its use allows for improving the accuracy of segmentation by increasing the number of layers and applying other techniques to improve the quality of the model [22]. This architecture has been used to select small extracellular vesicles on TEM images [23], to identify viral particles [24], and for multiclass segmentation of brain electron microscopy images [25]. Machine learning methods for the segmentation of microscopic images allow researchers to carry out their analysis quickly and accurately, which results in their high demand and the creation of a number of application packages, which do not require the user to have knowledge of programming and tuning of parameters [12,23]. All these properties facilitate the use of advanced microscopic image analysis by biologists and physicians in solving biological problems.

In the present work, we used a collection of TEM images of mouse melanoma tissue sections that were obtained and verified by experts in the field of ultrastructural studies. We used five deep learning network models based on the U-Net architecture and ResNet34 encoder for the segmentation of mitochondria and ER in these images. The downstream image analysis was implemented to access the mitochondria–ER contacts in the segmented images. The UltraNet web server was developed for publicly available automated analysis of mitochondria, ER, and MERCSs on TEM images.

## 2. Materials and Methods

### 2.1. Experimental Design

Male C57BL/6 mice of 10–12 weeks of age, with weights of 20–22 g, were used in the experiment. Skin melanoma B16 cell line was obtained from the Institute of Cytology and Genetics, Novosibirsk, Russia. B16 cells were subcutaneously injected into the right inguinal area of the mice (1 × 10^6^ cell). Ten days after tumor cell implantation, the mice were divided into experimental groups (*n* = 5 per group): (1) mice with intact tumors (control, MC); (2) mice receiving daily injections of brefeldin A (9.6 mg/kg, injected into the tumor periphery, MB [Tocris, Bristol, UK]); (3) mice treated daily with rapamycin (1.875 mg/kg, orally, MR [InvivoGen, Toulouse, France]); and (4) mice treated daily with both rapamycin and brefeldin A (MRB). Rapamycin, a known autophagy stimulator, induces the formation of autophagic structures by inhibiting the mTOR signaling pathway [26]. Brefeldin A disrupts the flow of proteins from the ER through the Golgi apparatus to the plasma membrane and interferes with normal sorting of membrane proteins, resulting in the accumulation of non-transportable proteins in the ER cisternae and activation of ER stress [27]. Animals were euthanized by craniocervical dislocation seven days after the start of drug administration.

### 2.2. Transmission Electron Microscopy

The tumor specimens were fixed with 4% paraformaldehyde and then incubated with 1% osmium tetroxide (OsO_4_) at 4 °C for 1 h. The fixed specimens were dehydrated with gradient alcohols and embedded in Epon. Next, 70–100 nm ultrathin sections were cut with a Leica EM UC 7 microtome (Leica Microsystems GmbH, Wetzlar, Germany) and stained with 1% uranyl acetate and lead citrate. Electron micrographs were taken at 25.000× at 80 kV with a JEM 1400 electron microscope (JEOL, Tokyo, Japan).

### 2.3. Image Stratification and Manual Segmentation

We used 296 TEM images of tumor sections obtained from 20 experimental animals. The image size was 2028 × 2048 pixels (pixel width = 0.38 nm). The images were divided into training, test, and validation samples so that each image was represented in only one of them. It was expected that the effects of the two drugs on cancer cells would lead to changes in cellular structure morphology, resulting in our sample set not being homogeneous. When training and testing the neural network model, we accounted for this by forming three variants of image stratification into training, validation, and test sets.

(1) Exclusive stratification: the experimental conditions for the images in the test set (MR) differ from those for the images in the training and validation sets (MC, MB, MRB).
(2) Inclusive Stratification: the training, validation, and test sets include images obtained under various conditions in approximately equal proportions.
(3) Control stratification: images of control samples (animal cancer cells without treatment) were used for training, validation, and testing.

The distribution of images across the sets for different types of stratification is shown in Table 1.

**Figure 1:**
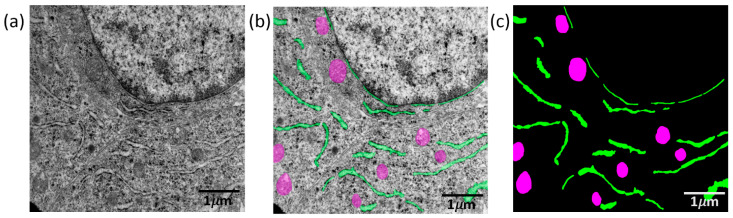
Result of manual segmentation of TEM images of tumor sections using the Hasty Data Annotation service. (**a**) Original image; (**b**) image with marked mitochondria (magenta) and ER (light green); (**c**) image mask, where colored areas correspond to mitochondria and ER, and black areas correspond to the background. Scale bars represented at the bottom right corner of images.

Image pixels were manually segmented into background and two classes (mitochondria, ER). The Hasty Data Annotation service (available at https://hasty.ai/, accessed on 20 March 2025) was used to mark up the images. Two expert annotators manually segmented the organelles using the Hasty.ai tool. One annotator (primary) created masks and a second (independent doctoral-level expert in cell biology) reviewed a subset for consistency. Discrepancies were resolved by consensus before model training. An example of a mask from the original image is shown in Figure 1: mitochondria appear as darker ovals 0.2–0.4 μm in width, with clear boundaries, and include on average 4–5% of the pixels in the image. ER is represented as the light areas of elongated irregular shape and include on average 2–4% of the image pixels. Images were analyzed without any modifications/preprocessing.

### 2.4. Network Architectures for Image Segmentation

Several deep learning networks based on the U-Net architecture [21] were used. This convolutional neural network topology is widely used for image segmentation in medical image analysis [28]. It consists of an encoder and decoder connected with each other [21]. Various modifications to the U-Net structure have been proposed recently to improve the semantic segmentation of biomedical images, which are classified into several groups [28]: skip connection enhancements, backbone design enhancements, bottleneck enhancements, transformers, rich representation enhancements, and probabilistic design. Several networks with different architectures, encoders, and initial weights were used for the comparison of their performance in the mitochondria/ER segmentation task. The list of network models and their specific features is provided in Table 2.

These networks differ from the basic model, U-Net-Vanilla, through the addition of various attention features (U-Net-scSE, MA-Net), the use of the feature pyramid approach to address multiscale problems (DeepLabV3+, U-Net-FPN), and the utilization of initial weights obtained from unsupervised pre-training (CEM500K-MoCoV2, CEM500K-SwAV). The first five networks are based on the ResNet34 encoder [29], which has demonstrated high accuracy in image classification tasks compared to others (VGG-19 and encoder with structure without shortcut connections) [29]. ResNet34 was chosen because it was trained on the images for the classification tasks and, therefore, it is good at selecting the features of the objects in the image, which helps to improve the segmentation accuracy.

**Figure 2:**
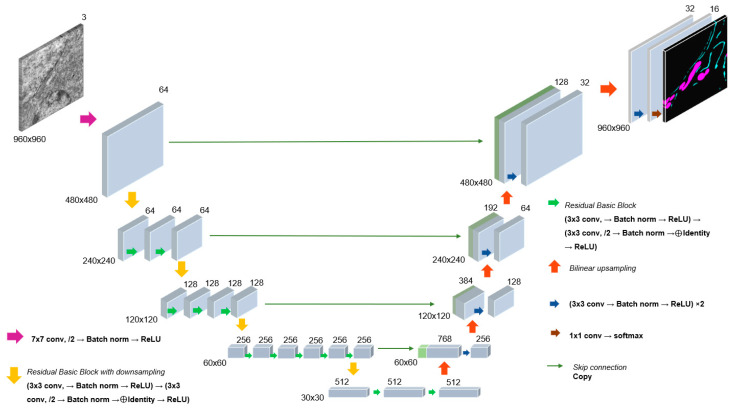
U-Net architecture based on Resnet34 encoder for mitochondria and endoplasmic reticulum recognition on TEM images of tumor sections.

The ResNet34 network architecture has 34 layers and is shown in Figure 2. The encoder consists of convolution layers, each performing convolution operations, normalization batch, ReLU activation functions, and max pooling operations. The encoder output is 512 × 30 × 30. The U-Net decoder consists of upsampling (reverse expansion), convolution layers, concatenation with corresponding encoder layers, and normalization. The outputs of each decoder layer are concatenated with the corresponding encoder layers of the same dimensionality; the last decoder layer is 2 × 960 × 960, which corresponds to a single-channel segmentation mask for each of the two predicted classes.

U-Net-Vanilla, U-Net-scSE, MA-Net, DeepLabV3+, and U-Net-FPN used ImageNet initial weights. The adaptive optimizer AdaBelief [36] was used to select network parameters. The initial learning rate was set to 10^−3^ with a batch size of 6. The combined loss function, DiceCE [37], which is defined as the sum of Cross Entropy [30] and Dice [38], was taken to optimize the model weights. Dice calculates the measure of similarity between the predicted mask and the true segmentation mask. Cross Entropy evaluates the quality of the classification. Based on a training sample of images and their corresponding masks, the network models were trained over 300 epochs for three types of dataset stratification.

CEM500K-MoCoV2 and CEM500K-SwAV are based on the ResNet50 encoder, and their initial weights were obtained through pre-training on a large-scale heterogeneous unlabeled cellular electron microscopy image dataset [35] (https://github.com/volume-em/cem-dataset, accessed on 14 April 2026). The weights for CEM500K-MoCov2 were obtained using the momentum contrast unsupervised learning algorithm [39], and the weights for CEM500K-SwAV were obtained using the same algorithm [40]. With these approaches, different views of the same image (positive pairs) are pulled together, while those corresponding to different images (negative pairs) are pushed apart. It was demonstrated that the CEM500K pre-trained models significantly outperformed randomly initialized models and models pre-trained on ImageNet when evaluated on several datasets of cellular electron microscopy images [35]. For these models, two SMP decoders, U-Net and FPN, were tested.

### 2.5. Network Model Training and Segmentation Accuracy Evaluation

To train the segmentation model based on the neural network, the open-source framework PyTorch v. 2.5.0 (Facebook, Menlo Park, CA, USA) was chosen. A computer with a GPU Nvidia RTX 2080ti was used to train the models. For U-Net-Vanilla, U-Net-scSE, MA-Net, DeepLabV3+, and U-Net-FPN, training parameters were the following: max_epochs = 400, train_size = 960, encoder_name = resnet34, train batch size = 2, validation batch size = 1.

The single grayscale channel input was used by the CEM500K-MoCoV2 and CEM500K-SwAV models. The mean and standard deviation values at the checkpoint were used for normalization. During training, the encoder weights of the CEM500K models were frozen, and the weights were changed only for the decoder and segmentation head (CEM fine-tuning configuration with finetune_layer = none). The default schedule was as follows: AdamW with weight_decay = 0.1 and OneCycleLR with 2500 optimizer steps at lr = 0.003, using mixed precision 16. The crop size was set to 960 px, and the batch size was set to 2 (see https://github.com/volume-em/cem-dataset, accessed on 14 April 2026).

The performance of the network model was evaluated using several metrics: the confusion matrix, precision, recall (sensitivity), *IoU* (also known as the Jaccard index), and the *F*1 measure (also known as the Dice similarity coefficient, *DSC*) [41,42]. The confusion matrix represents true positives (*TP*), false positives (*FP*), true negatives (*TN*), and false negatives (*FN*) for the comparison of objects in images segmented manually and by a machine learning algorithm. Other parameters could be obtained using these metrics [41,42]:

$$Precision\;=\frac{TP}{TP+FP+FN}\;,$$

$$Recall=Sensitivity=\frac{TP}{TP+FN}\;,$$

$$IoU=\frac{TP}{TP+FP+FN}\;,$$

$$F1=DSC=\frac{2\cdot TP}{2\cdot TP+FP+FN}\;.$$

The closer all these measures are to one, the better the performance of the algorithm is. All these metrics were calculated separately for the background, mitochondria, and endoplasmic reticulum. To estimate these metrics, pooling pixels from all images in the dataset was used. This appeared to be close to the estimate performance and the calculated values for each image separately, and was followed by taking their averages.

### 2.6. Augmentation

Augmentation of the training sample is a common method for improving the learning process of models, including deep neural networks. It implies the expansion of the training sample by creating new examples on the basis of the existing ones with the help of various distortions. Algorithms implemented in the Albumentations library [43] were used the for the image augmentation. Input crop sizes were set to 960 × 960 pixels. The same geometric and color augmentation was used for the U-Net-Vanilla, U-Net-scSE, MA-Net, DeepLabV3+, and U-Net-FPN models. For the CEM500K models, augmentation was performed using a single-channel grayscale input and normalization by the mean and standard deviation, based on parameters from the CEM500K checkpoint. The list of transformation parameters applied at different stages and for various network configurations is shown in Table S1 (Supplementary File).

### 2.7. Additional Image Datasets Used for Pre-Training Neural Networks

Three additional image datasets were used to evaluate the ability of the network models to differentiate cellular structures after pre-training. The first dataset, Lucchi++ [44,45], included images of mouse hippocampus cells acquired using focused ion beam scanning electron microscopy, taken from a 5 × 5 × 5 µm section of the hippocampus of the mouse brain. The whole image stack is 2048 × 1536 × 1065 vx, and manually created mitochondria segmentation masks are available for two neighboring image stacks (each 1024 × 768 × 1065 vx) [45]. This dataset includes 165 images for both training and testing. The second dataset, Kasthuri++ [44,46], included images of a sub-volume of the mouse neocortex acquired by serial section electron microscopy. The 3D images have volume dimensions of 1463 × 1613 × 85 vx (training set, 85 images) and 1334 × 1553 × 75 vx (test set, 75 images), with a resolution of 3 × 3 × 30 nm per voxel. Lucchi++ and Kasthuri++ datasets were obtained by re-annotation of the initial images to reduce misclassification [44]. The third dataset, DeepPI-EM (https://yoonlab.unist.ac.kr/index.php/research/mitochondria-tem-dataset/, accessed on 14 April 2026), included transmission electron microscopy (TEM) images of mouse skeletal muscle [47] (21 training and 6 test images) at a resolution of 2048 × 2048 pixels. Examples of images from the three datasets are shown in Figure S1 (Supplementary File). In total, 271 training samples and 246 test samples were used. It should be noted that all external datasets were annotated for the presence of mitochondria only and do not contain any information about the endoplasmic reticulum.

External data were used in two modes (Table 3): (1) pre-training of the U-Net-scSE and U-Net-FPN models; and (2) training of these models in combination with our data (Inclusive stratification; see Table 1). In the first case, training was performed using external data only; then, fine-tuning of the model weights was performed using the Inclusive stratification dataset. In the second case, images from the Inclusive stratification dataset were used in combination with external images; testing was performed on 74 Inclusive stratification test images.

### 2.8. Analysis of Segmented Images

The downstream analysis of segmented images included the estimation of the area of the mitochondria and ER in the image and determination of MERCs. Contours for mitochondria and ER in the images identified using the OpenCV v. 4.6.0.66 computer vision library [48]. For each contour, its area is estimated in nm^2^ and the total area occupied by mitochondria and ER is reported for the image.

**Figure 3:**
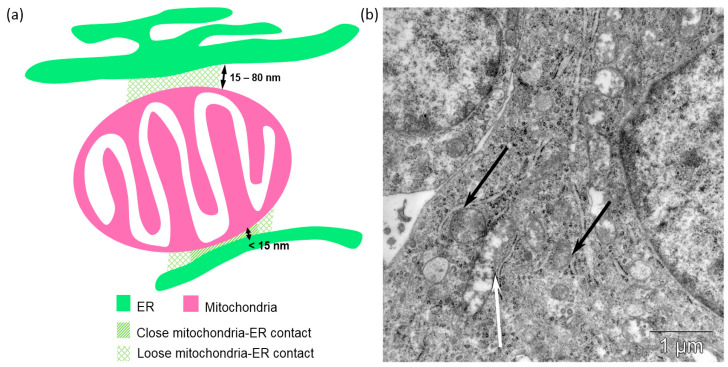
Mitochondria–ER contact sites (MERCs). (**a**) Schematic representation of first (close) and second (loose) types of MERCs. (**b**) The two types of MERCs in the TEM images of tumor sections (loose contact shown by white arrow; close contacts shown by black arrows).

To identify MERCs, we analyzed mitochondria contours. The mitochondria contour continuous segments participating in MERCs were identified as pixels less than 80 nm (211 pixels) away from the pixels of the ER contours. Additionally, we distinguished two types of MERCs: close contacts for pixels at distances smaller than 15 nm (first-type MERCs) and loose contacts otherwise (second-type MERCs). Contacts were classified as “close” if <15 nm, based on MERC widths reported around 10–15 nm [49]. For each type of MERCs, we estimated two characteristics: number of contacts and the total length of the mitochondria contour segments participating in MERCs (in nm). The second characteristics provide an estimate of the fraction of the mitochondria surface involved in MERCs. The analysis was performed using the OpenCV library. A schematic representation of the close and loose MERCs and corresponding contour regions are shown in Figure 3.

To assess how the accuracy of image segmentation affects the determination of MERC structural characteristics, we compared their evaluations for images manually segmented and automatically segmented using the aforementioned algorithm. The MERC characteristics included the number of first-type contacts (*N*_C1_), total length of first-type contacts (*L*_C1_), number of second-type contacts (*N*_C2_), and total length of second-type contacts (*L*_C2_). These two types of parameters were compared based on Pearson’s correlation coefficient.

### 2.9. Comparison of Estimates of the Area of Mitochondria and ER Obtained Automatically and Manually

Additionally, a comparison between the fraction of the image area occupied by mitochondria and ER estimated automatically in the images segmented by the neural network and manually was performed. To estimate the areas of image segments belonging to mitochondria or ER in the image manually, we applied a grid to an image using ImageJ 1.54r [50]. The grid size was 1024 × 1024, and the grid cell size was 170 nm. Grid vertices were marked with a label of two types depending on which region the given vertex fell into (mitochondrion or ER cistern). For each of the two classes, we calculated the ratio of the number of corresponding labels to the total number of vertices in the grid. This ratio is an estimate of the fraction of the image area occupied by the objects of a given class. In the case of automatic image processing, the fraction of the image area occupied by the mitochondria and ER was obtained directly using the total image area and estimates of the mitochondria and ER total areas in the image (see above).

The Pearson correlation coefficient was used to estimate the relationship between the area of objects in the image obtained manually and automatically.

Statistical analysis was performed using R language (Version 4.3.3) (http://www.r-project.org, accessed on 1 March 2025). Statistical tests used aggregated animal-level data to avoid analyzing images as independent samples. The normality of the data was evaluated using the Shapiro–Wilk test, with a significance criterion of *p* < 0.05. The Kruskal–Wallis test and Dunn post hoc test were carried out to compare four experimental groups. Two-tailed *p* < 0.05 was deemed to signify statistical significance.

### 2.10. UltraNet Web Server

**Figure 4:**
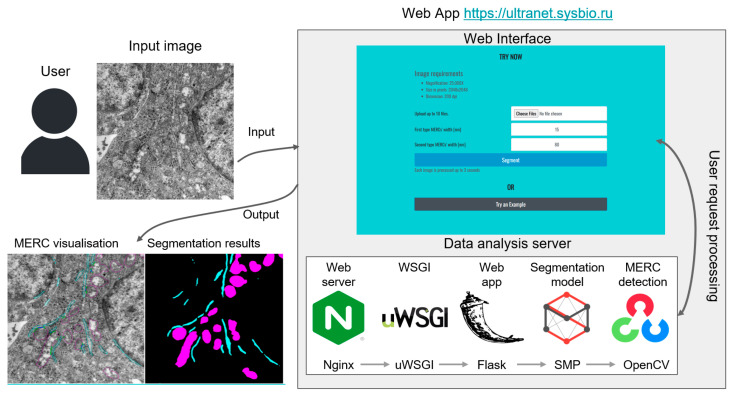
UltraNet server architecture. Two types of output images are generated: MERC visualization image (the mitochondria are shown in magenta, the ER is shown in turquoise) and image mask, where colored areas correspond to mitochondria and ER, and black areas correspond to the background.

The U-Net-scSE deep learning neural network model for cell structure segmentation in TEM images trained on Inclusive stratification without pre-training is implemented as the UltraNet web-service, available at https://ultranet.sysbio.ru, accessed on 20 January 2026. The web server architecture is shown in Figure 4. The service runs under Nginx 1.14.0 for load balancing. The uWSGI 2.0.20 module (available at https://github.com/unbit/uwsgi, accessed on 20 January 2025) handles communication between the server and the application. The user interface is implemented using Flask 2.0.3 (available at https://github.com/pallets/flask/, accessed on 20 January 2025). The Flask application handles user requests, generates static pages through the Jinja 3.0.3 templating engine (available at https://github.com/pallets/jinja/, accessed on 20 January 2025) and returns them to the user.

The U-Net model from the segmentation-models-pytorch 0.21 library (https://github.com/qubvel/segmentation_models, accessed on 20 January 2025) is used for cell structure segmentation. The segmented image analysis is performed using the OpenCV v. 4.6.0.66 library [48]. The whole system is deployed on a virtual machine under the Ubuntu 18.04.6 operating system. The processing time of one image on the server, including data input and output operations, is on average about 2 s.

## 3. Results

### 3.1. Assessing the Performance of the Segmentation Model

**Figure 5:**
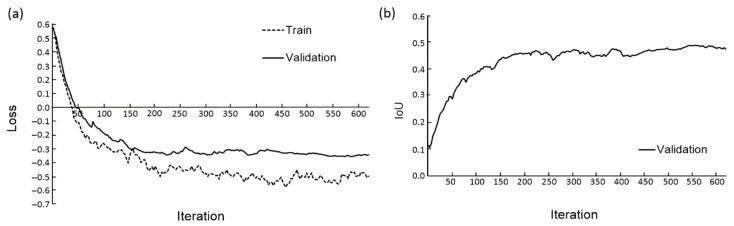
Graphs of the training of the network model trained using Inclusive stratification dataset. (**a**) The *Loss* function change during training for train and validation datasets. (**b**) The *IoU* value change during training for validation dataset.

The results of the U-Net-scSE neural network training for the Inclusive stratification data split are shown in Figure 5 as an example. During the training process, both the *Loss* and the *IoU* for the validation sample converge to stationary values starting from the 400th iteration (Figure 5a,b). The resulting model parameters were used to evaluate its quality on the test sample. The *IoU* value on the test dataset was 0.773 for the mitochondrial class and 0.592 for the ER class. Similar behavior of the loss and *IoU* parameters during the training process was observed for other stratification types.

Table 2 represents confusion matrices for the semantic segmentation of the test images in the Inclusive stratification dataset obtained by five neural network models. The initial weights were taken from ImageNet.

It is demonstrated in Table 4 that the segmentation performance for different models is close: the true positive predictions are highest for the background (0.977–0.982), and lower for mitochondria (0.789–0.798) and the endoplasmic reticulum (0.617–0.697). Most errors are caused by the misclassification of mitochondria and ER pixels as background. These errors are more prevalent for the ER (0.302–0.382) than for mitochondria (0.164–0.201). Other types of errors account for 1% or less.

Four performance metrics were estimated using test images from the Inclusive stratification dataset for these network models. The metrics were calculated for each class separately by pooling all pixels and then averaged separately for each class for each model. The results are presented in Table 5. The metric values are close for all networks, with differences of about several percent. However, the best performance for the *IoU* and *F*1 metrics across all classes, as well as for precision for the background and the endoplasmic reticulum, was demonstrated by the U-Net-scSE model. The average values for these metrics are also the highest. This suggests that, in general, the U-Net-scSE model outperforms the other models.

In terms of the average *IoU* metric, the U-Net-FPN model is ranked second, and the U-Net-Vanilla model is ranked third. In terms of the average *F*1 metric, the U-Net-Vanilla model is ranked second, and the U-Net-FPN model is ranked third.

Examples of image segmentation performed by the U-Net-scSE model, with high (0.770) and low (0.301) average *IoU* values for mitochondria and ER, are demonstrated in Figure S2 (Supplementary File). In these images, errors are caused by the misclassification of small darker areas as the endoplasmic reticulum (Figure S2a) or mitochondria (Figure S2b).

### 3.2. Assessing the Performance of the Segmentation Models Utilizing External Data for Training

Two models, U-Net-scSE and U-Net-FPN, were used to check whether the utilization of microscopic images from external datasets (Lucchi++, Kasthuri++, and DeepPy-EM) improves segmentation. Two training methods were applied. In the first case, the network model was pre-trained using external images only, followed by tuning of the model parameters using the Inclusive stratification dataset. In the second case, the models were trained using a mixture of external images and training/validation images from the Inclusive stratification dataset.

In both cases, the models were evaluated using the Inclusive stratification test images. Performance metrics were estimated separately for each class, as described above. The results are presented in Table 6 and demonstrate that neither the *IoU* nor the *F*1 metrics surpass the values obtained by the U-Net-scSE model without pre-training or the use of external images (see Table 5). For some models and training configurations, higher *Precision* and *Recall* values were achieved compared to those of the U-Net-scSE model. These values were obtained using the pre-training protocol for both architectures (U-Net-scSE and U-Net-FPN). Interestingly, the highest values for the *IoU* and *F*1 metrics in this experiment were obtained for the U-Net-FPN architecture, regardless of whether pre-training was used or a combination of our data and external data was employed during training.

### 3.3. Assessing the Performance of the Pre-Trained CEM500K Models

Neural network models based on the ResNet50 architecture, pre-trained using the CEM500K EM image dataset by two methods (MoCoV2 or SwAV) [35], were fine-tuned using our Inclusive stratification training and validation images. These models were used with two decoders, U-Net and FPN. After fine-tuning, the models were tested on the Inclusive stratification test dataset. The results are presented in Table 7. The table demonstrates that neither metric surpasses the values obtained by the U-Net-scSE model (see Table 5). In this experiment, however, better performance was demonstrated by the CEM500K-MoCoV2/FPN model.

### 3.4. Performance of U-Net-scSE Model on Various Stratifications and Groups of Animals

U-Net-scSE model was evaluated using two additional stratifications, exclusive and control (see Table 1). The confusion matrices evaluated on test data for this experiment are presented in Table 8. In general, the model demonstrated lower performance on these datasets, in comparison with the results for Inclusive stratification (see Table 4).

For all stratifications, the primary classification error is predicting mitochondria and ER as background. This error is approximately 1.5–2 times higher for ER than for mitochondria.

The *IoU* values for background, mitochondrial, and ER pixels in the test dataset from the Inclusive stratification for images from different experiments are provided in Table 9.

Table 9 demonstrates that the segmentation accuracy of pixels belonging to the three classes varies across samples from different experiments. However, there is no experiment for which the proportion of correctly classified background, mitochondrial, and ER pixels is consistently higher than for the others. For cells from animals treated with brefeldin (MB), the pixels of ER are most accurately identified among all other classes, while the other classes are the least accurately identified. For cells from animals treated with rapamycin (MR), the best accuracy is observed for background pixel identification. For cells from animals treated with both rapamycin and brefeldin (MRB), the highest proportion of correctly predicted mitochondrial pixels (0.945) is achieved, but the proportion of correctly identified ER pixels is the lowest (0.679). Overall, the data presented in Table 9 align with the general evaluation of the confusion matrix for the Inclusive stratification, as shown in Table 4: the highest accuracy is achieved for background pixels, followed by mitochondrial and ER pixels.

### 3.5. Accuracy of Length and Quantity Estimation of MERCs

The assessments provided above give an overview of the algorithm’s effectiveness in the segmentation task. However, for researchers, the final analysis involves evaluating the structural characteristics of the cells, particularly MERCs. A comparison of the contact characteristics of mitochondria and ER obtained from manual and automatic image segmentation is presented in Table 10. It provides Pearson’s correlation coefficients between the two sets of parameter estimates.

In the first case, the contact distance is shorter, and classification errors for pixels are more pronounced. Nevertheless, in both the first and second cases, the correlation coefficients are significantly different from 0 and exceed 0.4.

### 3.6. Visualization of Analysis Results

**Figure 6:**
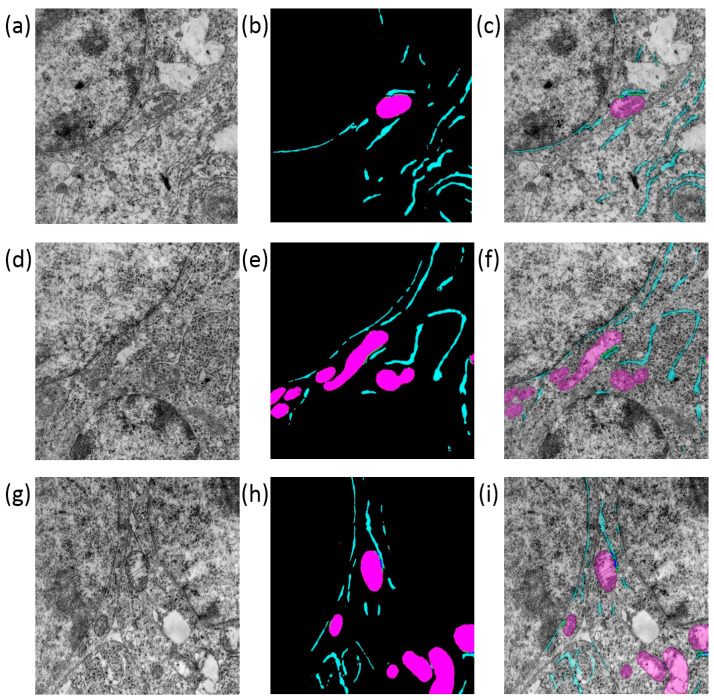
Examples of segmentation of three different images of mouse melanoma tumor sections using neural network and manual markup: (**a**,**d**,**g**) original images; (**b**,**e**,**h**) neural network segmentation results; (**c**,**f**,**i**) manual image markup. The mitochondria are shown in magenta, the ER is shown in turquoise.

The results of the UltraNet segmentation for three images from the test sample are shown in Figure 6. The figure demonstrates good agreement between the results of automatic and manual image segmentation. The location of ER and mitochondria regions are highly similar and the differences occur mainly for the borders between the background and cell structures.

**Figure 7:**
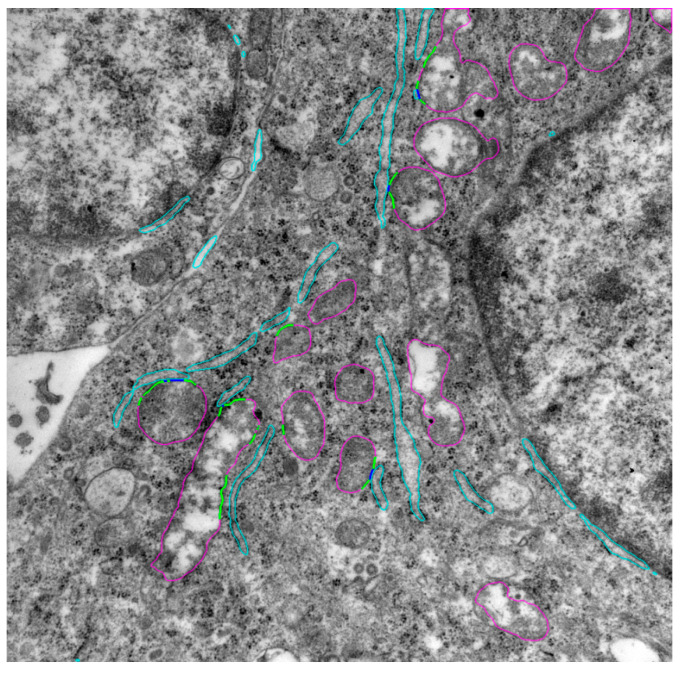
Example of the result of determining the contours for mitochondria, ER, and the contact sites between them. The colors correspond to the UltraNet output: mitochondrial contours are shown in magenta; ER contours are shown in turquoise. Regions of close contact on mitochondrial contours are shown in blue color; distant ones are shown in green.

Examples of contacts between ER and mitochondria identified by UltraNet are shown in Figure 7. The following estimates were obtained for the image: the number of mitochondria is 15; the mitochondrial area is 2,707,933 nm^2^; the ER area is 777,175 nm^2^; the number of first-type MERCs is 5; the total length of the first-type MERC contour segments is 380 nm; the number of second-type MERCs is 10; the total length of second-type MERC contour segments is 2446 nm.

### 3.7. Comparison of Manual and Automated Estimate of the Cell Structure Area

**Figure 8:**
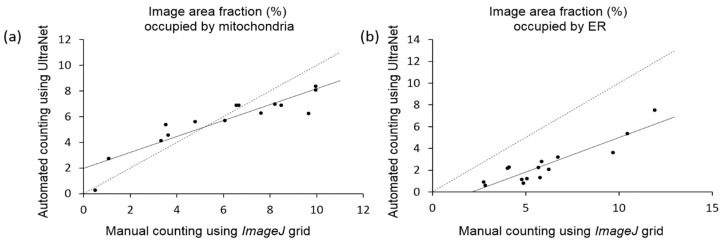
Comparison of the manual (X axis) and UltraNet (Y axis) estimates of the fraction (in %) of the mitochondria and ER area in the image. (**a**) Fraction of the image area occupied by mitochondria; (**b**) fraction of the image area occupied by ER. The regression lines between *x* and *y* are solid in two panels; the *y* = *x* lines are dashed.

Figure 8 demonstrates a scatterplot of the estimates of the area fraction occupied by mitochondria and ER obtained using manual counting and UltraNet.

The Pearson correlation coefficients between the fraction of the image area calculated manually and automatically for mitochondria and ER are identical (0.91). However, the regression lines for these scatterplots deviate from the *y* = *x* line remarkably. For the mitochondria (Figure 8a), the manual analysis in comparison with the automatic one yields smaller area values within the 0–4% interval and larger values within the 8–11% interval. For the ER area, the manual estimation gives systematically higher area values compared to the automatic ones (Figure 8b). These discrepancies can be easily explained by the fact that the minimal area unit for the manual counting is the grid cell area. For thin ER contours and small mitochondria, this method will always overestimate the area values. For large-sized mitochondria, on the contrary, there is a situation when part of the object extends beyond the cell boundaries but does not include the neighboring nodes. In this case, manual counting gives an underestimate of the object areas (image area fraction > 8%; Figure 8a).

It should be noted that manual estimation of the image area occupied by mitochondria and ER usually takes several minutes, and it is difficult for a human to perform such image analysis for a long time. The UltraNet estimates are determined by the contour itself and do not depend on the grid dimension. It is performed in a very short time.

### 3.8. UltraNet Application for Quantitative Analysis of Tumor Cells Ultrastructure

To demonstrate the practical effectiveness of the proposed image analysis, we use it for a comparative analysis of the cell ultrastructure parameters for tissue samples from the control and experimentally treated animals with rapamycin as an autophagy modulator [26] and brefeldin A as an ER stress modulator [27], as well as their combination. The data comprise all 296 TEM images (Table 1) segmented by UltraNet with parameters obtained using Inclusive stratification. MERC evaluation algorithms (see Section 2.8: Analysis of Segmented Images) were applied for downstream processing of segmented images. The results are presented in Table 11. It demonstrates that administering animals with rapamycin, brefeldin A, and their combination yields a significant increase in the ER area (almost double) in comparison with control animals. There was an increase by a factor of ~1.4 in the mitochondrial area in the group treated with brefeldin A. Additionally, a significant increase in the length of type 1 MERCs in the groups treated with brefeldin A compared to the groups treated with rapamycin was observed. The length of type 2 MERCs also increased in MB in comparison with MR, albeit this was insignificant. Other parameters did not show any significant differences in the MR, MB, and MRB groups compared to the control.

### 3.9. UltraNet Server Interface

**Figure 9:**
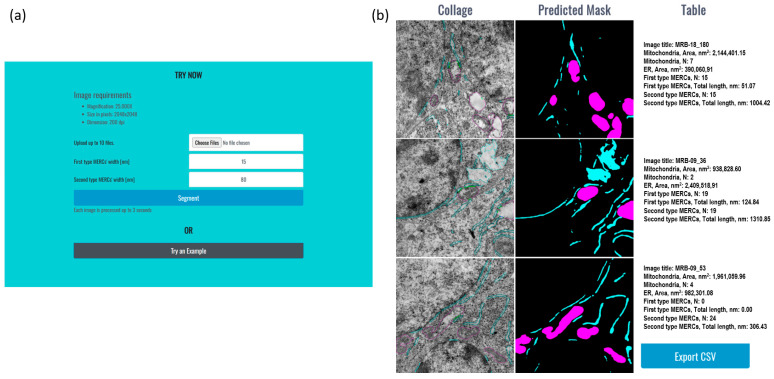
The UltraNet web-service interface. (**a**) The input form; (**b**) the output page (the mitochondria are shown in magenta, the ER is shown in turquoise).

The UltraNet web-service input page contains several elements (Figure 9a):

- Image uploading field;
- Two numerical fields for thresholds for close and loose contact determination;
- Button for sending the data;
- Button to execute analysis of example images.

The UltraNet output represents a set of rows. The number of rows is equal to the number of input images. Each row contains (1) an input image with mitochondria and ER contours and MERCs marked by different colors, (2) the image segmentation mask, and (3) the information about detected organelles and their contacts. The result page contains a button to save the image analysis statistics in csv format (Figure 9b).

As an initial practical check of accessibility, two users without programming experience independently completed the upload-and-analysis workflow and successfully obtained segmentation outputs from TEM images without assistance. This observation supports the practical usability of the current interface for exploratory use; however, it should be interpreted as an informal pilot assessment rather than a formal usability study.

## 4. Discussion

The segmentation of structures on TEM biological/medical images remains a challenging task requiring considerable human control and correction [6,7,12,13]. Modern research is based on the analysis of hundreds of TEM images [51]; therefore, automatic methods are of great importance in this field. Algorithms based on deep learning neural networks have been developed over the last decade with promising results [10,19,23]. For example, the method based on the U-Net deep learning architecture allowed for the identification of extracellular vesicles (EVs) with a Jaccard coefficient (*IoU*) up to 0.88 for correctly detected objects [23]. Nikishin et al. [19] developed a method to detect EVs in TEM images and obtained mean average precision values between 0.817 and 0.86 depending on the bounding box *IoU* threshold. A similar performance estimate was obtained for mitochondria identification in EM images using deep learning. For example, Casser et al. [44] performed image segmentation into mitochondria and background for the re-annotated Lucchi image dataset [45] using modified U-Net architecture. The authors demonstrated a high efficiency in mitochondria identification, with the Jaccard index between 0.845 and 0.90. Several models of the deep learning networks based on the U-Net were applied for semantic segmentation of the mitochondria with *IoU* values ranging from 0.55 to 0.77 depending on the training/testing datasets [52]. Conrad and Narayan [35] developed the MitoNet model based on the Panoptic-DeepLab architecture and image post-processing for mitochondria recognition in 2D/3D EM images and estimated its performance on images from different cell types (fly brain, HeLa cells, glycolytic muscle, etc.). The *IoU* measure varied in this work from 0.315 to 0.899 depending on the dataset.

Some authors have performed tissue EM image segmentation into sets of classes corresponding to several organelles. Shaga Devan et al. [10] performed semantic segmentation of biological EM images into cytoplasm, nucleus, and background using an approach based on ensemble prediction with several deep learning networks. They demonstrated that the ensemble approach on seven datasets yields Jaccard index values between 0.72 and 0.99 and outperforms the U-Net model. Gallusser et al. [53] implemented a 3D U-Net segmentation model for the identification of mitochondria, ER, Golgi apparatus, nuclear pores, and clathrin-coated vesicles in 3D beam scanning electron microscopy images. They achieved an *F*1 measure for voxel classification from 0.74 to 0.95 depending on the training/testing dataset combination. Their analysis also demonstrated better performance in identifying mitochondria in comparison with other organelles. Heinrich et al. [54] presented an automated procedure for the segmentation of whole cellular organelles from FIB-SEM data. From the results, the scientists also estimated the contact sites between organelles. The authors trained a 3D U-Net model and achieved an *F*1 value of 0.967 for mitochondria and 0.841 for the ER of cultured HeLa cells.

Here, several models with different architectures based on the U-Net topology were tested to perform semantic segmentation of TEM images of murine skin melanoma cells. These models included the basic U-Net-Vanilla architecture, models utilizing various attention blocks (U-Net-scSE, MA-Net), and models using blocks capturing multilevel image structure (DeepLabV3+, U-Net-FPN). The analysis of the test images demonstrated that the best performance (*IoU* and *F*1 measures) was achieved by the U-Net-scSE model. The *IoU*/*F*1 values of 0.773/0.872 and 0.593/0.744 were obtained for mitochondria and ER segmentation, respectively. These values are within the range of *F*1 performance estimates for mitochondria and ER obtained in studies of different cell types by Heinrich et al. [54]. The performance metrics for mitochondria segmentation using our best model (U-Net-scSE) are generally lower than those reported in other works, where *IoU* values for mitochondria were obtained by other authors using different datasets [44,47,52]. These differences may be caused by the fact that two classes of areas in the image are recognized in our work. On the other hand, in our images (Figure S2, Supplementary File), compared with third-party datasets (Figure S1, Supplementary File), the mitochondrial areas are much less clearly defined. In particular, some darkened areas may be mistaken by our model for mitochondria (Figure S2b, Supplementary File).

Interestingly, the performance of the ER identification is lower in comparison to mitochondria, similar to other groups’ [53] results. This is likely because mitochondria have a large size and specific inner membrane structure with clearly visible double membrane boundaries. Determining the ER is more challenging. Unlike mitochondria, the boundaries of the ER in tumor cells are distinguishable mainly due to ribosomes localized on the membrane of the ER cisternae, but which may be absent from some parts of the membrane. We suggest that the low *IoU* value for ER is most likely due to the fact that the network model does not recognize smooth ER membranes.

The U-Net-scSE model achieves comparable (but lower) performance when using two additional stratifications of images. These stratifications differentiate images obtained from treated and non-treated mice in different ways (Table 1). In the Exclusive stratification, images from mice treated with rapamycin were combined in the test set, while the other images were used as training/validation data. In the Control stratification, images from control mice were used for testing. On the contrary, in the Inclusive stratification, images from treated and untreated mice were split into all three subsets (training, validation, and test). This allows variations in the mitochondria and ER structures, occurring due to the treatment of mice with drugs, to be taken into account (see also Table 9). In general, these comparisons demonstrate the high generalization ability of the U-Net-scSE model on our data.

Additional tests were conducted to evaluate neural network pre-training using external image datasets, utilizing two strategies (training and tuning or combining external images with ours during training). The results demonstrated that some strategies can increase *Precision* and *Recall* values compared to the U-Net-scSE model without pre-training. However, neither strategy provided a performance improvement in terms of *IoU* and *F*1 metrics. Two additional networks pre-trained using the CEM500K EM image dataset [35] were tested here after fine-tuning on the training/validation images from the Inclusive stratification dataset. Again, no performance improvement was detected in comparison with the U-Net-scSE model.

Previously, the utility of using pre-trained models in the segmentation of EM images was demonstrated using various datasets [35,47]. However, pre-trained models may not always improve segmentation performance. For example, it was demonstrated by Conrad and Narayan that pre-trained models only slightly improve *IoU* metrics for the Lucchi++ and Kasthuri++ datasets [35]. It was noted that the efficiency of pre-training might depend on the underlying biology or tissue architecture and similarities in the image acquisition and sample preparation protocols. It is likely that imaging protocols and the specific structural features of our images (see Figures S1 and S2 in the Supplementary File) may contribute to the weak efficiency of pre-trained neural networks for our image datasets.

Our work is similar in terms of aim to the analysis performed by Lui et al. [55]. They presented an automatic 3D reconstruction of mitochondria, ER, and their contacts in neurons from scanning electron microscopy data of the mouse cerebral cortex. They used the Mask R-CNN network model for the mitochondria and ResNet50 model for the ER identification. Two models were trained independently. The post-processing step allowed for the detection of contacts between segmented mitochondria and ER in 3D. The authors achieved an accuracy value of 0.8021 on the Jaccard index (*IoU*) for mitochondria identification (the performance of the ER segmentation was not estimated). In our work, we achieved a comparable performance using a single network model for both mitochondria and ER in 2D image analysis. Our UltraNet application also performs a post-processing step to identify MERCs and collect related statistics. Additionally, our model enables the segmentation of images acquired by transmission electron microscopy, which, in contrast to scanning electron microscopy, is less labor-intensive and more commonly employed in routine biological research.

Methods of the cell EM image analysis using deep learning provide fast and accurate identification of organelles and their morphometry. However, most of them are implemented as Python v.2.5.0 packages and require users to have programming skills [35,52,53]. This makes it difficult for biologists and medics who are not qualified in programming to use such approaches for solving biological problems. For the wider use of the developed models, software tools with a simple interface are created, which do not require programming knowledge [19,23,56]. In this paper, a solution in the form of a web-service is proposed for analyzing TEM images. The user can only upload an image and obtain the analysis results as a file.

Image analysis by deep learning algorithms requires datasets of large size. Good practice involves making datasets publicly available for other studies. Several such datasets were suggested in the field of cellular structure analysis by EM imaging [35,44,57,58,59]. We made our annotated TEM image dataset of mouse melanoma tumor sections available for public access. We hope these data will help to improve TEM image analysis algorithms in future work by other researchers. By lowering the technical barrier to TEM image analysis, UltraNet may be particularly useful for biologists who require rapid first-pass quantification of mitochondrial morphology, ER area, and MERCs but do not have dedicated programming or image analysis expertise. In practice, such a tool can facilitate hypothesis generation, prioritize images for expert review, and improve the consistency of routine morphometric measurements across experiments. We therefore view UltraNet as a decision-support and throughput-enhancing platform rather than as a total replacement for expert ultrastructural interpretation.

Collectively, our findings demonstrate the utility and simplicity of the UltraNet platform for assessing MERCs in tumor cells using B16 skin melanoma model. The accumulation of misfolded proteins within the ER lumen during ER stress activates the unfolded protein response (UPR), thereby promoting ER membrane remodeling and the formation of MERCs [60]. The intensity of the stressor significantly influences the UPR’s adaptability and the temporal dynamics of the response: prolonged UPR signaling and maladaptive ER stress can enhance mitochondrial calcium uptake and trigger apoptosis.

Brefeldin A disrupts ER-to-Golgi protein trafficking and is a well-established inducer of ER stress [61]. The marked increase in ER area observed in MB cells is therefore consistent with brefeldin A-induced perturbation of ER homeostasis. MERCs are major sites of ER–mitochondria Ca^2+^ exchange and stress signaling, and adaptive ER stress responses can enhance ER–mitochondria coupling and mitochondrial Ca^2+^ import [62,63].

A recent study by [64] showed that the ER–mitochondria distance is a critical parameter for efficient Ca^2+^ transfer and mitochondrial oxidative metabolism, with an optimal calcium flux observed at an inter-organelle distance near 20 nm. In this context, the increase in both the number and the length of close MERCs in MB cells may reflect stress-associated remodeling of the ER–mitochondria interface. By contrast, rapamycin does not simply oppose brefeldin A-induced stress; mTORC1 inhibition can also reshape ER–mitochondria coupling, but with a different spatial and functional profile and in close connection with autophagy-related adaptation [62]. The intermediate phenotype observed in the MRB group is therefore compatible with partial modulation of the brefeldin A-driven stress response rather than with a purely additive interaction. Collectively, these findings support the interpretation that brefeldin A primarily promotes ER stress-associated MERC remodeling, whereas rapamycin may modify this response through mTOR- and autophagy-dependent pathways [62].

The functional roles of MERCs and proteins involved in ER–mitochondria Ca^2+^ signaling, such as the mitochondrial calcium uniporter and inositol 1,4,5-trisphosphate (IP3) receptors, are only beginning to be elucidated [65]. Understanding MERC dynamics has important therapeutic implications in cancer due to MERCs regulating fundamental cellular processes including calcium signaling, lipid metabolism, apoptosis, and mitochondrial function, all of which are frequently dysregulated in cancer cells. Recent literature highlights MERCs as promising targets for therapeutic intervention to enhance cancer treatment outcomes [66,67,68,69,70]. Therefore, these data underscore the importance of analyzing MERCs in tumor cells and developing tools to monitor their dynamics.

MERC detection in the present study was operationally defined from two-dimensional TEM images by measuring the minimal distance between segmented mitochondrial and ER contours. This approach is grounded in electron microscopy, which remains the gold standard for identifying inter-organelle contacts, but it does not fully capture the three-dimensional continuity of MERCs [71]. Consequently, contact frequency and length may be affected both by the sectioning plane and by segmentation inaccuracies near organelle boundaries. We therefore interpret the resulting metrics as robust 2D estimates within the current imaging setting rather than as exhaustive measurements of MERC architecture in three dimensions. Contacts were further subclassified as “close” when the ER–mitochondria distance was <15 nm, which was used as a conservative operational threshold based on literature indicating that tighter MERCs often occur in the ~10–20 nm range, while the broader MERC spacing reported across studies spans approximately 10–80 nm [1,72].

A major limitation of the present study is the restricted biological scope of the training and test data. The model was developed using 296 TEM images from a single tumor type, and its performance has therefore been demonstrated primarily within this imaging domain. Although transfer learning and data augmentation can improve segmentation accuracy in microscopy applications with limited annotated data, models trained on narrowly defined datasets may still be sensitive to domain shifts related to tissue type, organelle morphology, sample preparation, staining, fixation, or microscope settings. Future studies should include external validation on independent datasets acquired from additional cell types, tissues, and imaging workflows.

## 5. Conclusions

For the automatic identification of mitochondria and ER organelles in TEM images of mouse melanoma, we developed a deep learning network model based on the U-Net architecture and ResNet34 encoder. To train and test the model, we collected a dataset of 206 TEM images of mouse melanoma tissue sections annotated by experts. Here, five models of the U-Net segmentation network, including the basic U-Net-Vanilla architecture; models utilizing various attention blocks; and models using blocks capturing multilevel image structure, were evaluated for the segmentation of mitochondria and the endoplasmic reticulum. The best performance on the test dataset was demonstrated by the U-Net-scSE network. Two models were tested for their ability to utilize pre-training with external images (Lucchi++, Kasthuri++, and DeepPi-EM). Additionally, models pre-trained using the CEM500K dataset were tested after fine-tuning the parameters on our data. The results demonstrated that pre-training or the use of pre-trained networks did not improve the *IoU* and *F*1 metrics on our test dataset.

The downstream analysis of segmented images was implemented to access the mitochondria–ER contacts. We implemented our algorithms as the UltraNet web server for automated analysis of mitochondria, ER, and mitochondria–ER contacts on TEM images. The processing time of one image on the server, including data input and output operations, is on average about 2 s. Our results demonstrated the high efficiency of the deep learning neural networks to identify mitochondria, ER, and MERCs using TEM images and provide the possibility of such analysis for a large number of researchers.

In conclusion, UltraNet provides an accessible proof-of-principle workflow for the automated segmentation of mitochondria and endoplasmic reticulum and for downstream MERC quantification in TEM images acquired under conditions similar to those of the present study. The current results demonstrate the feasibility of a web-based approach for routine ultrastructural analysis within this imaging domain, but they do not yet establish universal applicability across tissues, cell types, or sample preparation protocols. Broader adoption will require additional external validation on independent datasets and prospective benchmarking in diverse experimental settings.

## Figures and Tables

**Table 1:** Image distribution by experiments and datasets for three types of stratification.

Experiment	Images per Experiment	Stratification								
		Exclusive			Inclusive			Control		
		Training	Validation	Testing	Training	Validation	Testing	Training	Validation	Testing
MC	102	87	15	0	52	25	25	60	21	21
MB	31	31	0	0	15	8	8	0	0	0
MR	102	0	0	102	50	26	26	0	0	0
MRB	61	6	55	0	31	15	15	0	0	0
Total	296	124	70	102	148	74	74	60	21	21
%	100	42	24	34	50	25	25	58	21	21

**Table 2:** Description of network models used in the work.

Model	Architecture	Encoder/Initial Weights	Description	References
U-Net-Vanilla	U-Net with basic convolutions	ResNet-34/ImageNet	U-Net architecture with basic 2D convolutions and skipped connections between encoder and decoder	[29,30]
U-Net-scSE	U-Net with scSE-attention block in decoder	ResNet-34/ImageNet	U-Net with skipped connections between encoder and decoder and concurrent spatial and channel squeeze and excitation block	[29,30,31]
MA-Net	Multi-scale attention Net	ResNet-34/ImageNet	U-Net with position-wise attention block to model spatial dependencies between pixels in the bottleneck feature maps with self-attention	[29,32]
DeepLabV3+	DeepLabV3+	ResNet-34/ImageNet	U-Net architecture with atrous spatial pyramid pooling in the bottleneck and decoder module to recover the object boundaries	[29,33]
U-Net-FPN	U-Net with feature pyramid network decoder	ResNet-34/ImageNet	Top-down feature pyramid architecture with lateral connections combining shallow features and deep semantic information	[29,34]
CEM500K-MoCoV2	ResNet50	ResNet50/CEM500K, MoCoV2 method	U-Net models with ResNet50 encoder and unsupervised pre-training using CEM500K image dataset and MoCoV2 method	[35]
CEM500K-SwAV	ResNet50	ResNet50/CEM500K, SwAV method	Segmentation U-Net models with ResNet50 encoder and unsupervised pre-training using CEM500K image dataset and SwAV method	[35]

**Table 3:** Utilizing external data images in two modes during network model training and testing.

External Dataset Usage Mode	Train	Validation	Test
Pre-training	271 external train images	246 external test images	Not used
Combined	148 Inclusive stratification train images + 271 external train images	74 Inclusive stratification validation images + 246 external test images	74 Inclusive stratification test images

**Table 4:** Confusion matrices for the segmentation of images into the background, mitochondria, and the endoplasmic reticulum, obtained for the test image dataset and various models using the Inclusive stratification dataset. The highest values of true positive predictions for each class are shown in bold.

Model	True\Predicted	Background	Mitochondria	ER
U-Net-Vanilla	Background	**0.982**	0.008	0.010
	Mitochondria	0.189	0.796	0.001
	ER	0.365	0.001	0.635
U-Net-scSE	Background	**0.982**	0.007	0.010
	Mitochondria	0.191	0.795	0.001
	ER	0.302	0.000	**0.697**
MA-Net	Background	0.977	0.011	0.012
	Mitochondria	0.201	**0.798**	0.000
	ER	0.382	0.000	0.617
DeepLabV3+	Background	0.981	0.008	0.012
	Mitochondria	0.197	0.789	0.001
	ER	0.376	0.000	0.624
U-Net-FPN	Background	0.981	0.007	0.012
	Mitochondria	0.164	0.795	0.000
	ER	0.319	0.001	0.680

**Table 5:** Performance metrics for five network models on the test image dataset from Inclusive stratification. Largest values in the same metrics/class are shown in bold.

Model	Class	*IoU*	*Precision*	*Recall*	*F*1
U-Net-Vanilla	Background	0.963	**0.983**	0.979	0.981
	Mitochondria	0.764	0.860	0.872	0.866
	ER	0.567	0.700	**0.749**	0.723
	Average	0.764	0.848	**0.867**	0.857
U-Net-scSE	Background	**0.966**	**0.983**	**0.983**	**0.983**
	Mitochondria	**0.773**	0.862	0.881	**0.872**
	ER	**0.593**	0.758	0.731	**0.744**
	Average	**0.777**	**0.868**	0.865	**0.866**
MA-Net	Background	0.959	0.978	0.981	0.979
	Mitochondria	0.732	**0.863**	0.828	0.845
	ER	0.525	0.697	0.681	0.689
	Average	0.739	0.846	0.830	0.838
DeepLabV3+	Background	0.961	0.981	0.979	0.980
	Mitochondria	0.761	0.853	0.876	0.865
	ER	0.536	0.692	0.704	0.698
	Average	0.753	0.842	0.853	0.848
U-Net-FPN	Background	0.964	0.981	0.982	0.982
	Mitochondria	0.772	0.856	**0.887**	0.871
	ER	0.558	0.746	0.689	0.716
	Average	0.765	0.861	0.853	0.856

**Table 6:** Performance metrics for two network models pre-trained using external image data on the test images from Inclusive stratification. Largest values in the same metrics/class are shown in bold. Metrics that outperform U-Net-scSE for the same class are underlined.

Model/Training Method	Class	*IoU*	*Precision*	*Recall*	*F*1
U-Net-scSE/Pre-training	Background	0.962	0.982	0.980	0.981
	Mitochondria	0.747	0.856	0.854	0.855
	ER	0.578	0.712	**0.754**	0.733
	Average	0.762	0.850	0.863	0.856
U-Net-FPN/Pre-training	Background	**0.964**	0.979	**0.985**	**0.982**
	Mitochondria	**0.767**	**0.884**	0.853	**0.868**
	ER	0.552	**0.747**	0.679	0.711
	Average	0.761	**0.870**	0.839	0.854
U-Net-scSE/Combined datasets	Background	0.962	0.981	0.980	0.981
	Mitochondria	0.765	0.861	**0.873**	0.867
	ER	0.544	0.699	0.710	0.705
	Average	0.757	0.847	0.855	0.852
U-Net-FPN/Combined datasets	Background	0.963	0.982	0.980	0.981
	Mitochondria	0.750	0.851	0.864	0.857
	ER	**0.584**	0.723	0.752	**0.737**
	Average	**0.766**	0.852	**0.865**	**0.858**

**Table 7:** Performance metrics for CEM500K pre-trained models on the test images from Inclusive stratification. Largest values in the same metrics/class are shown in bold.

Model/Decoder	Class	*IoU*	*Precision*	*Recall*	*F*1
CEM500K-MoCoV2/U-Net	Background	0.940	0.956	0.982	0.969
	Mitochondria	0.534	0.837	0.596	0.696
	ER	0.356	0.612	0.460	0.525
	Average	0.610	0.802	0.679	0.730
CEM500K-MoCoV2/FPN	Background	0.958	**0.981**	0.976	0.978
	Mitochondria	**0.712**	0.801	**0.865**	**0.832**
	ER	**0.547**	0.709	**0.706**	**0.707**
	Average	**0.739**	0.830	**0.849**	**0.839**
CEM500K-SwAV/U-Net	Background	0.938	0.963	0.973	0.968
	Mitochondria	0.570	0.670	0.767	0.726
	ER	0.335	0.705	0.390	0.502
	Average	0.614	0.786	0.710	0.732
CEM500K-SwAV/FPN	Background	0.959	0.975	**0.983**	**0.979**
	Mitochondria	0.706	0.874	0.786	0.828
	ER	0.531	0.709	0.678	0.693
	Average	0.732	0.853	0.816	0.833

**Table 8:** Confusion matrices for segmentation of images into background, mitochondria, and endoplasmic reticulum (ER) for the test image dataset for U-Net model for three types of stratification data. Diagonal elements are shown in bold.

Stratification	True\Predicted	Background	Mitochondria	ER
Exclusive	Background	**0.990**	0.005	0.005
	Mitochondria	0.279	**0.721**	0.000
	ER	0.435	0.00	**0.565**
Control	Background	**0.981**	0.015	0.004
	Mitochondria	0.253	**0.747**	0.000
	ER	0.440	0.000	**0.560**

**Table 9:** *IoU* values for classified pixels for images from different groups of animals on the test set. Inclusive stratification data split was used. The highest values in each column are highlighted in bold, and the lowest values are in italics.

Experiment	Background	Mitochondria	ER
MB	*0.970*	*0.620*	**0.790**
MC	0.978	0.852	0.686
MR	**0.989**	0.736	0.688
MRB	0.983	**0.945**	*0.679*

**Table 10:** Pearson’s correlation coefficients between MERC characteristics obtained from manual and automatic image segmentation. All values are significantly different from 0 (*p* < 0.01).

Stratification	First-Type MERCs		Second-Type MERCs	
	*N*_C1_	*L*_C1_, nm	*N*_C2_	*L*_C2_, nm
Exclusive	0.579	0.429	0.924	0.953
Inclusive	0.468	0.577	0.861	0.863
Control	0.625	0.528	0.912	0.949

**Table 11:** The results of intracellular structures and mitochondria–ER contact (MERC) analysis in tumor cells from animals with B16 melanoma in the control group (MC) and in animals treated with brefeldin A (MB) or rapamycin (MR) or their combination (MRB) using the UltraNet service. Type I MERCs were defined as contacts < 15 nm (C1), while type II MERCs were defined as contacts > 15 nm (C2). Data are presented as median/interquartile range as well as results of the Kruskal–Wallis test and Dunn’s post hoc test for comparison of the four experimental groups.

Parameter	MC	MB	MR	MRB
Mitochondria area, nm^2^	256,807.1/ 242,359.6	364,672.9/ 295,668.1	267,717.7/ 202,561.2	233,675.2/ 226,288.3
Number of mitochondria	5/5	5/2.5	5/4	5/4
ER area, nm^2^	70,802/ 87,196.2	140,556.8 ^1^/ 185,927.3	111,732.6 ^1^/ 95,019.1	132,819.6 ^1^/ 115,785.1
*N*_C1_	1/1	1/2.5	0/1	1/2
*L*_C1_, nm	12.5/77.1	50.5 ^2^/223.6	0/40.3	23/90.2
*N*_C2_	4/6	6/5.5	5/5.8	5/7
*L*_C2_, nm	587.6/887.6	889.5/831.8	684.2/898.3	663.6/795.3

*N*—number, *L*—length; ^1^ *p* < 0.05 (vs. control group); ^2^ *p* < 0.05 (vs. rapamycin group).

## Data Availability

The original data presented in the study are openly available in UltraNet at https://ultranet.sysbio.ru (accessed on 20 March 2026).

## Supplementary Materials

The following supporting information can be downloaded at https://www.mdpi.com/article/10.3390/jimaging12050215/s1: Table S1; Augmentation, preprocessing operations, and parameters used for training/validation/inference. Figure S1: Examples of typical images from external datasets. (a) Lucchi ++, mask0016.png, 1024x768 px; (b) Kasthuri++, mask1079.png, 1463x1613 px; (c) DeepPI-EM, x_5.tif, 2560x2560 px. Some mitochondria are shown in the images with arrows. Figure S2: Examples of segmentation of cell structures segmentation in TEM images of murine skin melanoma cells using U-Net-scSE model. Original image is on the left, manual label map is in the middle and predicted label map is on the right. In the label maps, the mitochondria are shown in magenta, the ER is shown in turquoise. (a). MP-09_4.tif image, average IoU for mitochondria and ER is 0.770; (b). MP-09_32.tif image, average IoU for mitochondria and ER is 0.301.

## Abbreviations

The following abbreviations are used in this manuscript:

EM	Electron microscopy
ER	Endoplasmic reticulum
EV	Extracellular vesicle
*IoU*	Intersection over union metric
MB	Mice receiving daily injections of brefeldin A
MC	Mice with intact tumors
MERCs	Mitochondria–endoplasmic reticulum contact sites
MR	Mice treated daily with rapamycin
MRB	Mice treated daily with both rapamycin and brefeldin A
ROS	Reactive oxygen species
TEM	Transmission electron microscopy
UPR	Unfolded protein response

## References

[1] Giacomello M., Pellegrini L. (2016). The Coming of Age of the Mitochondria–ER Contact: A Matter of Thickness. Cell Death Differ..

[2] Aoyama-Ishiwatari S., Hirabayashi Y. (2021). Endoplasmic Reticulum–Mitochondria Contact Sites—Emerging Intracellular Signaling Hubs. Front. Cell Dev. Biol..

[3] Simoes I.C.M., Morciano G., Lebiedzinska-Arciszewska M., Aguiari G., Pinton P., Potes Y., Wieckowski M.R. (2020). The Mystery of Mitochondria-ER Contact Sites in Physiology and Pathology: A Cancer Perspective. Biochim. Biophys. Acta (BBA)-Mol. Basis Dis..

[4] Sassano M.L., Van Vliet A.R., Agostinis P. (2017). Mitochondria-Associated Membranes As Networking Platforms and Regulators of Cancer Cell Fate. Front. Oncol..

[5] An G., Park J., Song J., Hong T., Song G., Lim W. (2024). Relevance of the Endoplasmic Reticulum-Mitochondria Axis in Cancer Diagnosis and Therapy. Exp. Mol. Med..

[6] Harris J.R. (2015). Transmission Electron Microscopy in Molecular Structural Biology: A Historical Survey. Arch. Biochem. Biophys..

[7] Lam J., Katti P., Biete M., Mungai M., AshShareef S., Neikirk K., Garza Lopez E., Vue Z., Christensen T.A., Beasley H.K. (2021). A Universal Approach to Analyzing Transmission Electron Microscopy with ImageJ. Cells.

[8] Papadopulos F., Spinelli M., Valente S., Foroni L., Orrico C., Alviano F., Pasquinelli G. (2007). Common Tasks in Microscopic and Ultrastructural Image Analysis Using ImageJ. Ultrastruct. Pathol..

[9] Bell C.G., Treder K.P., Kim J.S., Schuster M.E., Kirkland A.I., Slater T.J.A. (2022). Trainable Segmentation for Transmission Electron Microscope Images of Inorganic Nanoparticles. J. Microsc..

[10] Shaga Devan K., Kestler H.A., Read C., Walther P. (2022). Weighted Average Ensemble-Based Semantic Segmentation in Biological Electron Microscopy Images. Histochem. Cell Biol..

[11] Yildirim B., Cole J.M. (2021). Bayesian Particle Instance Segmentation for Electron Microscopy Image Quantification. J. Chem. Inf. Model..

[12] Kotrbová A., Štěpka K., Maška M., Pálenik J.J., Ilkovics L., Klemová D., Kravec M., Hubatka F., Dave Z., Hampl A. (2019). TEM ExosomeAnalyzer: A Computer-Assisted Software Tool for Quantitative Evaluation of Extracellular Vesicles in Transmission Electron Microscopy Images. J. Extracell. Vesicles.

[13] Rangayyan R.M., Kamenetsky I., Benediktsson H. (2010). Segmentation and Analysis of the Glomerular Basement Membrane in Renal Biopsy Samples Using Active Contours: A Pilot Study. J. Digit. Imaging.

[14] Proença M.C., Nunes J.F.M., De Matos A.P.A. (2013). Texture Indicators for Segmentation of Polyomavirus Particles in Transmission Electron Microscopy Images. Microsc. Microanal..

[15] Kylberg G., Uppström M., Hedlund K.-O., Borgefors G., Sintorn I.-M. (2012). Segmentation of Virus Particle Candidates in Transmission Electron Microscopy Images: Segmentation of Virus Particle Candidates in TEM Images. J. Microsc..

[16] Calvino J.J., López-Haro M., Muñoz-Ocaña J.M., Puerto J., Rodríguez-Chía A.M. (2022). Segmentation of Scanning-Transmission Electron Microscopy Images Using the Ordered Median Problem. Eur. J. Oper. Res..

[17] Arganda-Carreras I., Kaynig V., Rueden C., Eliceiri K.W., Schindelin J., Cardona A., Sebastian Seung H. (2017). Trainable Weka Segmentation: A Machine Learning Tool for Microscopy Pixel Classification. Bioinformatics.

[18] Horwath J.P., Zakharov D.N., Mégret R., Stach E.A. (2020). Understanding Important Features of Deep Learning Models for Segmentation of High-Resolution Transmission Electron Microscopy Images. npj Comput. Mater..

[19] Nikishin I., Dulimov R., Skryabin G., Galetsky S., Tchevkina E., Bagrov D. (2021). ScanEV—A Neural Network-Based Tool for the Automated Detection of Extracellular Vesicles in TEM Images. Micron.

[20] Sadre R., Ophus C., Butko A., Weber G.H. (2021). Deep Learning Segmentation of Complex Features in Atomic-Resolution Phase-Contrast Transmission Electron Microscopy Images. Microsc. Microanal..

[21] Falk T., Mai D., Bensch R., Çiçek Ö., Abdulkadir A., Marrakchi Y., Böhm A., Deubner J., Jäckel Z., Seiwald K. (2019). U-Net: Deep Learning for Cell Counting, Detection, and Morphometry. Nat. Methods.

[22] Iglovikov V., Shvets A. (2018). TernausNet: U-Net with VGG11 Encoder Pre-Trained on ImageNet for Image Segmentation. arXiv.

[23] Gómez-de-Mariscal E., García-López-de-Haro C., Ouyang W., Donati L., Lundberg E., Unser M., Muñoz-Barrutia A., Sage D. (2021). DeepImageJ: A User-Friendly Environment to Run Deep Learning Models in ImageJ. Nat. Methods.

[24] Matuszewski D.J., Sintorn I.-M. (2019). Reducing the U-Net Size for Practical Scenarios: Virus Recognition in Electron Microscopy Images. Comput. Methods Programs Biomed..

[25] Getmanskaya A.A., Sokolov N.A., Turlapov V.E. (2022). Multiclass U-Net Segmentation of Brain Electron Microscopy Data Using Original and Semi-Synthetic Training Datasets. Program. Comput. Softw..

[26] Li J., Kim S.G., Blenis J. (2014). Rapamycin: One Drug, Many Effects. Cell Metab..

[27] Moon J.L., Kim S.Y., Shin S.W., Park J.-W. (2012). Regulation of Brefeldin A-Induced ER Stress and Apoptosis by Mitochondrial NADP+-Dependent Isocitrate Dehydrogenase. Biochem. Biophys. Res. Commun..

[28] Azad R., Aghdam E.K., Rauland A., Jia Y., Avval A.H., Bozorgpour A., Karimijafarbigloo S., Cohen J.P., Adeli E., Merhof D. (2024). Medical Image Segmentation Review: The Success of U-Net 2022. IEEE Trans. Pattern Anal. Mach. Intell..

[29] He K., Zhang X., Ren S., Sun J. (2016). Deep Residual Learning for Image Recognition. Proceedings of the 2016 IEEE Conference on Computer Vision and Pattern Recognition (CVPR).

[30] Ronneberger O., Fischer P., Brox T., Navab N., Hornegger J., Wells W.M., Frangi A.F. (2015). U-Net: Convolutional Networks for Biomedical Image Segmentation. Medical Image Computing and Computer-Assisted Intervention—MICCAI 2015.

[31] Roy A.G., Navab N., Wachinger C. (2018). Concurrent Spatial and Channel Squeeze & Excitation in Fully Convolutional Networks. International Conference on Medical Image Computing and Computer-Assisted Intervention.

[32] Fan T., Wang G., Li Y., Wang H. (2020). MA-Net: A Multi-Scale Attention Network for Liver and Tumor Segmentation. IEEE Access.

[33] Chen L.-C., Zhu Y., Papandreou G., Schroff F., Adam H., Ferrari V., Hebert M., Sminchisescu C., Weiss Y. (2018). Encoder-Decoder with Atrous Separable Convolution for Semantic Image Segmentation. Computer Vision—ECCV 2018.

[34] Lin T.-Y., Dollar P., Girshick R., He K., Hariharan B., Belongie S. (2017). Feature Pyramid Networks for Object Detection. Proceedings of the 2017 IEEE Conference on Computer Vision and Pattern Recognition (CVPR).

[35] Conrad R., Narayan K. (2021). CEM500K, a Large-Scale Heterogeneous Unlabeled Cellular Electron Microscopy Image Dataset for Deep Learning. eLife.

[36] Zhuang J., Tang T., Ding Y., Tatikonda S., Dvornek N., Papademetris X., Duncan J.S. (2020). AdaBelief Optimizer: Adapting Stepsizes by the Belief in Observed Gradients. Adv. Neural Inf. Process. Syst..

[37] Taghanaki S.A., Zheng Y., Kevin Zhou S., Georgescu B., Sharma P., Xu D., Comaniciu D., Hamarneh G. (2019). Combo Loss: Handling Input and Output Imbalance in Multi-Organ Segmentation. Comput. Med. Imaging Graph..

[38] Drozdzal M., Vorontsov E., Chartrand G., Kadoury S., Pal C., Carneiro G., Mateus D., Peter L., Bradley A., Tavares J.M.R.S., Belagiannis V., Papa J.P., Nascimento J.C., Loog M., Lu Z. (2016). The Importance of Skip Connections in Biomedical Image Segmentation. Deep Learning and Data Labeling for Medical Applications.

[39] Chen X., Fan H., Girshick R., He K. (2020). Improved Baselines with Momentum Contrastive Learning. arXiv.

[40] Caron M., Misra I., Mairal J., Goyal P., Bojanowski P., Joulin A. (2020). Unsupervised Learning of Visual Features by Contrasting Cluster Assignments. Adv. Neural Inf. Process. Syst..

[41] Taha A.A., Hanbury A. (2015). Metrics for Evaluating 3D Medical Image Segmentation: Analysis, Selection, and Tool. BMC Med. Imaging.

[42] Müller D., Soto-Rey I., Kramer F. (2022). Towards a Guideline for Evaluation Metrics in Medical Image Segmentation. BMC Res. Notes.

[43] Buslaev A., Iglovikov V.I., Khvedchenya E., Parinov A., Druzhinin M., Kalinin A.A. (2020). Albumentations: Fast and Flexible Image Augmentations. Information.

[44] Casser V., Kang K., Pfister H., Haehn D. (2020). Fast Mitochondria Detection for Connectomics. Proc. Mach. Learn. Res..

[45] Lucchi A., Smith K., Achanta R., Knott G., Fua P. (2012). Supervoxel-Based Segmentation of Mitochondria in EM Image Stacks with Learned Shape Features. IEEE Trans. Med. Imaging.

[46] Kasthuri N., Hayworth K.J., Berger D.R., Schalek R.L., Conchello J.A., Knowles-Barley S., Lee D., Vázquez-Reina A., Kaynig V., Jones T.R. (2015). Saturated Reconstruction of a Volume of Neocortex. Cell.

[47] Jang C., Lee H., Yoo J., Yoon H. (2025). Deep Learning-Driven Automated Mitochondrial Segmentation for Analysis of Complex Transmission Electron Microscopy Images. Sci. Rep..

[48] Bradski G., Kaehler A. (2008). Learning OpenCV: Computer Vision with the OpenCV Library.

[49] Lu X., Gong Y., Hu W., Mao Y., Wang T., Sun Z., Su X., Fu G., Wang Y., Lai D. (2022). Ultrastructural and Proteomic Profiling of Mitochondria-Associated Endoplasmic Reticulum Membranes Reveal Aging Signatures in Striated Muscle. Cell Death Dis..

[50] Schneider C.A., Rasband W.S., Eliceiri K.W. (2012). NIH Image to ImageJ: 25 Years of Image Analysis. Nat. Methods.

[51] Bock D.D., Lee W.-C.A., Kerlin A.M., Andermann M.L., Hood G., Wetzel A.W., Yurgenson S., Soucy E.R., Kim H.S., Reid R.C. (2011). Network Anatomy and in Vivo Physiology of Visual Cortical Neurons. Nature.

[52] Franco-Barranco D., Pastor-Tronch J., González-Marfil A., Muñoz-Barrutia A., Arganda-Carreras I. (2022). Deep Learning Based Domain Adaptation for Mitochondria Segmentation on EM Volumes. Comput. Methods Programs Biomed..

[53] Gallusser B., Maltese G., Di Caprio G., Vadakkan T.J., Sanyal A., Somerville E., Sahasrabudhe M., O’Connor J., Weigert M., Kirchhausen T. (2023). Deep Neural Network Automated Segmentation of Cellular Structures in Volume Electron Microscopy. J. Cell Biol..

[54] Heinrich L., Bennett D., Ackerman D., Park W., Bogovic J., Eckstein N., Petruncio A., Clements J., Pang S., Xu C.S. (2021). Whole-Cell Organelle Segmentation in Volume Electron Microscopy. Nature.

[55] Liu J., Li L., Yang Y., Hong B., Chen X., Xie Q., Han H. (2020). Automatic Reconstruction of Mitochondria and Endoplasmic Reticulum in Electron Microscopy Volumes by Deep Learning. Front. Neurosci..

[56] Belevich I., Jokitalo E. (2021). DeepMIB: User-Friendly and Open-Source Software for Training of Deep Learning Network for Biological Image Segmentation. PLoS Comput. Biol..

[57] Lucchi A., Li Y., Smith K., Fua P., Fitzgibbon A., Lazebnik S., Perona P., Sato Y., Schmid C. (2012). Structured Image Segmentation Using Kernelized Features. Computer Vision—ECCV 2012.

[58] Wei D., Lin Z., Franco-Barranco D., Wendt N., Liu X., Yin W., Huang X., Gupta A., Jang W.-D., Wang X., Martel A.L., Abolmaesumi P., Stoyanov D., Mateus D., Zuluaga M.A., Zhou S.K., Racoceanu D., Joskowicz L. (2020). MitoEM Dataset: Large-Scale 3D Mitochondria Instance Segmentation from EM Images. Medical Image Computing and Computer Assisted Intervention—MICCAI 2020.

[59] Gerhard S., Funke J., Martel J., Cardona A., Fetter R. (2013). Segmented Anisotropic ssTEM Dataset of Neural Tissue; Dataset.

[60] Casas-Martinez J.C., Samali A., McDonagh B. (2024). Redox Regulation of UPR Signalling and Mitochondrial ER Contact Sites. Cell. Mol. Life Sci..

[61] Carew J.S., Nawrocki S.T., Krupnik Y.V., Dunner K., McConkey D.J., Keating M.J., Huang P. (2006). Targeting Endoplasmic Reticulum Protein Transport: A Novel Strategy to Kill Malignant B Cells and Overcome Fludarabine Resistance in CLL. Blood.

[62] Bravo-Sagua R., López-Crisosto C., Parra V., Rodriguez-Peña M., Rothermel B.A., Quest A.F.G., Lavandero S. (2016). mTORC1 Inhibitor Rapamycin and ER Stressor Tunicamycin Induce Differential Patterns of ER-Mitochondria Coupling. Sci. Rep..

[63] Kunja C., Kumar V., Kodam P., Gopu C.D., Maity S. (2025). ER Stress Sensors at the ER-Mitochondrial Interface, Controlling Mitochondrial Health in Neurodegenerative Diseases. Front. Neurosci..

[64] Dematteis G., Tapella L., Casali C., Talmon M., Tonelli E., Reano S., Ariotti A., Pessolano E., Malecka J., Chrostek G. (2024). ER-Mitochondria Distance Is a Critical Parameter for Efficient Mitochondrial Ca^2+^ Uptake and Oxidative Metabolism. Commun. Biol..

[65] Bustos G., Ahumada-Castro U., Silva-Pavez E., Puebla A., Lovy A., Cardenas J.C. (2021). The ER-Mitochondria Ca^2+^ Signaling in Cancer Progression: Fueling the Monster. International Review of Cell and Molecular Biology.

[66] De Ridder I., Kerkhofs M., Lemos F.O., Loncke J., Bultynck G., Parys J.B. (2023). The ER-Mitochondria Interface, Where Ca^2+^ and Cell Death Meet. Cell Calcium.

[67] Fernandez Garcia E., Paudel U., Noji M.C., Bowman C.E., Rustgi A.K., Pitarresi J.R., Wellen K.E., Arany Z., Weissenrieder J.S., Foskett J.K. (2023). The Mitochondrial Ca^2+^ Channel MCU Is Critical for Tumor Growth by Supporting Cell Cycle Progression and Proliferation. Front. Cell Dev. Biol..

[68] Pang M., Yu L., Li X., Lu C., Xiao C., Liu Y. (2024). A Promising Anti-Tumor Targeting on ERMMDs Mediated Abnormal Lipid Metabolism in Tumor Cells. Cell Death Dis..

[69] Endoni B.T., Koval O.M., Allamargot C., Kortlever T., Qian L., Sadoski R.J., Juhr D., Grumbach I.M. (2025). MIRO1 Is Required for Dynamic Increases in Mitochondria-ER Contact Sites and Mitochondrial ATP During the Cell Cycle. Cells.

[70] Ziegler D.V., Parashar K., Leal-Esteban L., López-Alcalá J., Castro W., Zanou N., Martinez-Carreres L., Huber K., Berney X.P., Malagón M.M. (2025). CDK4 Inactivation Inhibits Apoptosis via Mitochondria-ER Contact Remodeling in Triple-Negative Breast Cancer. Nat. Commun..

[71] Benhammouda S., Vishwakarma A., Gatti P., Germain M. (2021). Mitochondria Endoplasmic Reticulum Contact Sites (MERCs): Proximity Ligation Assay as a Tool to Study Organelle Interaction. Front. Cell Dev. Biol..

[72] Wilson E.L., Metzakopian E. (2021). ER-Mitochondria Contact Sites in Neurodegeneration: Genetic Screening Approaches to Investigate Novel Disease Mechanisms. Cell Death Differ..

