# Supplementary material for: Cell Structure Segmentation in TEM Images of Murine Skin Melanoma Cells by Deep Learning Model
Source: J Imaging. 2026 May 18;12(5):215. doi: 10.3390/jimaging12050215 (PMC13208365; doi:10.3390/jimaging12050215)
Supplement: Supplementary file 1 [file jimaging-12-00215-s001.zip › jimaging-4214972-supplementary.pdf]

Supplementary file for Genaev et al.

Table S1. Augmentation, preprocessing operations and parameters used for training/validation/inference.

Stage	Operation	Parameters and their values	Probability of applying the transform
Train	PadIfNeeded before cropping	min_height=960, min_width=960; border_mode=BORDER_REFLECT101; value=0; mask_value=0	always
Train	RandomCrop	height=960, width=960	1.0
Train	PadIfNeeded before stride encoder	pad_height_divisor=32, pad_width_divisor=32; border_mode=BORDER_REFLECT101; value=0; mask_value=0	always
Train	HorizontalFlip	Albumentations library default settings	0.5
Train	VerticalFlip	Albumentations library default settings	0.5
Train	RandomRotate90	Rotation at angles that are multiples of 90 degrees	0.5
Train	Distortion, OneOf	ElasticTransform(p=0.3), GaussianBlur(p=0.3), GaussNoise(p=0.3), OpticalDistortion(p=0.3), GridDistortion(p=0.1), PiecewiseAffine(p=0.3)	block p=0.3
Train	Color transform for RGB images, OneOf	HueSaturationValue(hue_shift_limit=15, sat_shift_limit=25, val_shift_limit=0), CLAHE(clip_limit=2), RandomBrightnessContrast(brightness_limit=0.3, contrast_limit=0.3)	block p=0.3
Train	Color transform for grayscale CEM500K models, OneOf	CLAHE(clip_limit=2), RandomBrightnessContrast(brightness_limit=0.3, contrast_limit=0.3)	block p=0.3
Train	Normalization techniques	RGB input: Albumentations Normalize with default values; CEM500K models grayscale input: Normalize(mean=[checkpoint mean], std=[checkpoint std]); ToTensorV2(transpose_mask=True)	always
Validation / inference	PadIfNeeded before stride encoder	pad_height_divisor=32, pad_width_divisor=32; border_mode=BORDER_REFLECT101; value=0; mask_value=0	always
Validation / inference	Normalization techniques	RGB input: Albumentations Normalize with default values; CEM500K models grayscale input: Normalize(mean=[checkpoint mean], std=[checkpoint std]); ToTensorV2	always

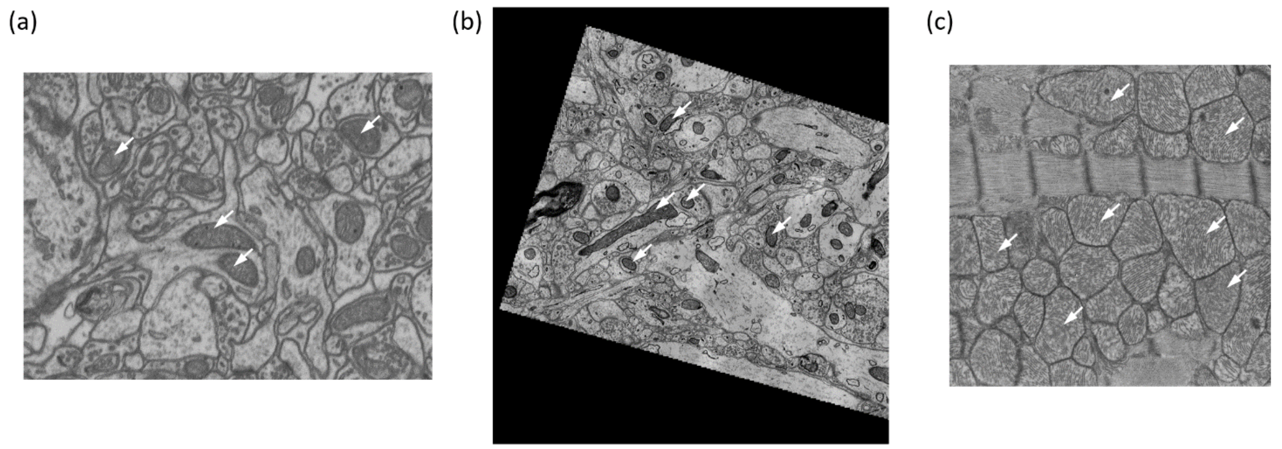

Figure S1. Examples of typical images from external datasets. a) Lucchi ++, mask0016.png, 1024x768 px; b) Kasthuri++, mask1079.png, 1463x1613 px; c) DeepPI-EM, x_5.tif, 2560x2560 px. Some mitochondria are shown in the images with arrows.

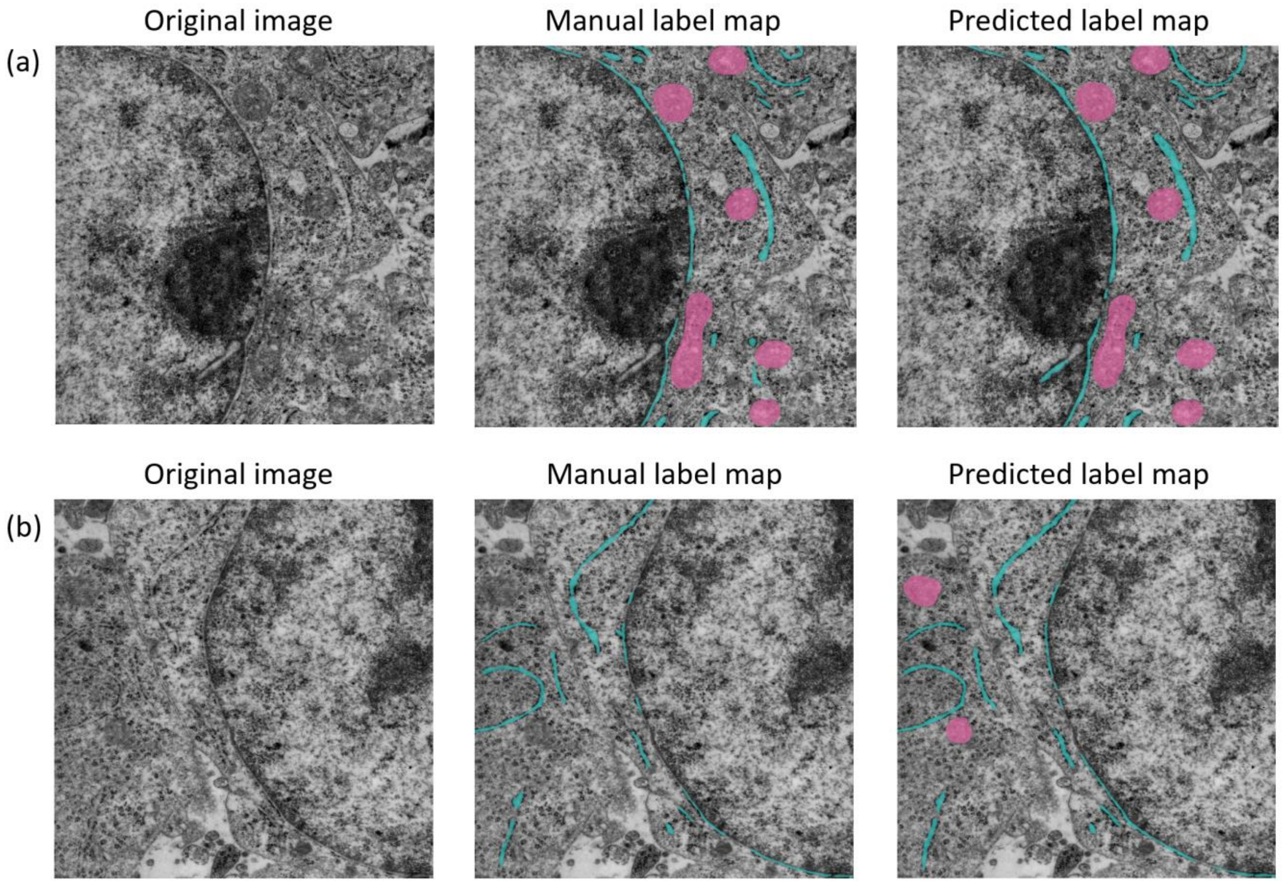

Figure S2. Examples of segmentation of cell structures segmentation in TEM images of murine skin melanoma cells using U-Net-scSE model. Original image is on the left, manual label map is in the middle and predicted label map is on the right. In the label maps, the mitochondria are shown in magenta, the ER is shown in turquoise. a). MP-09_4.tif image, average IoU for mitochondria and ER is 0.770; b). MP-09_32.tif image, average IoU for mitochondria and ER is 0.301.
